# Discovery of an alien species of mayfly in South America (Ephemeroptera)

**DOI:** 10.3897/zookeys.399.6680

**Published:** 2014-04-08

**Authors:** Frederico F. Salles, Jean-Luc Gattolliat, Kamila B. Angeli, Márcia R. De-Souza, Inês C. Gonçalves, Jorge L. Nessimian, Michel Sartori

**Affiliations:** 1 Laboratório de Sistemática e Ecologia de Insetos, Universidade Federal do Espírito Santo, Departamento de Ciências Agrárias e Biológicas, 29933-415, São Mateus, ES, Brasil; 2 Museum of Zoology, Palais de Rumine, Place Riponne 6, CH-1005 Lausanne, Switzerland; 3 Universidade Federal do Rio de Janeiro (UFRJ), Instituto de Biologia, Departamento de Zoologia, Laboratório de Entomologia, Caixa Postal 68044, Cidade Universitária, 21941-971, Rio de Janeiro, RJ, Brasil; 4 Programa de pós-graduação em Ciências Biológicas, modalidade Zoologia da UFRJ/Museu Nacional; 5 Instituto Oswaldo Cruz (Fiocruz), Departamento de Entomologia, Núcleo de Morfologia e Ultraestrutura de Vetores, 21045-900, Rio de Janeiro, RJ, Brasil; 6 Programa de Pós-Graduação em Biologia Animal, Universidade Federal Rural do Rio de Janeiro (UFRRJ), Seropédica, RJ, Brasil

**Keywords:** Aquatic insects, Ephemeroptera, invasive species, Baetidae, *Cloeon*, Neotropics

## Abstract

Despite its wide, almost worldwide distribution, the mayfly genus *Cloeon* Leach, 1815 (Ephemeroptera: Baetidae) is restricted in the Western hemisphere to North America, where a single species is reported. In the Neotropics, except for some species wrongly attributed to the genus in the past, there are no records of *Cloeon*. Recently, however, specimens of true *Cloeon* were collected along the coast of Espírito Santo, Southeastern Brazil. In order to verify the hypothesis that this species was recently introduced to Brazil, our aim was to identify the species based on morphological and molecular characters and to confirm the presence of true representatives of the genus in the Neotropics. Our results revealed that the specimens found in Brazil belong to the Afrotropical species *C. smaeleni* Lestage, 1924. The identity of the species, its distribution, along with its previous absence in regularly sampled sites, is a clear sign that the specimens of *C. smaeleni* found in Espírito Santo are introduced, well established, and that the colonization took place very recently.

## Introduction

Aquatic insects, despite their dominance in terms of diversity in most freshwater ecosystems, show a disproportional low number of invasive species when compared to other freshwater macroinvertebrates (Karatayev et al. 2009). Among entirely aquatic insect orders, there are no Plecoptera or Megaloptera and very few Trichoptera that are invasive (de Moor 1992). In mayflies (Ephemeroptera), a single case is documented, a Southeast Asia lentic species of the family Caenidae introduced accidentally in Hawaii during WWII (Zimmermann 1957) and now well established (Smith 2000). Among the necessary characteristics for invasive species, Kleunen et al. (2010) mentioned phenotypic plasticity, ability for uniparental reproduction, and fast growth in disturbed habitats. Besides that, de Moor (1992) mentioned some important characteristics in the case of invasive aquatic insects, such as: the generalist feeding, e.g. detritivores, year-round breeding capacities, the ability to colonize peri-urban environment and artificial waterbodies, and the climatic similarity of invaded and source environments. The ability to colonize water bodies with low level of oxygen, such as polluted streams and rivers or lentic habitats, can also be added to the characteristics mentioned by de Moor (1992). Tolerant species are much more likely to find a suitable site, and pass through physiological filters, than those with high requirements (see Karatayev et al. 2009).

Due to their diversity, abundance and role in nutrient cycling, mayflies are a critical component in freshwater ecosystems throughout the world, and most species are good bioindicators of the water quality (Menetrey et al. 2008; Brittain and Sartori 2009). Around 3500 species are known over the world, the vast majority colonizing running waters (Barber-James et al. 2008). Some species, however, are capable of colonizing lentic habitats, eutrophic water bodies, and present most of the aforementioned requirements for potential invaders.

The genus *Cloeon* Leach, 1815 (Ephemeroptera) is one of the most common and most diversified genera of mayflies. It encompasses 75 species with 24 of them in the Palaearctic realm, 23 in the Afrotropical and 20 in the Oriental. It is also present, though less diversified, in the other realms, except the Neotropics where true *Cloeon* have never been reported. Because of its long imaginal stage in females, ca 14 days for mated (Degrange 1960) to 28 days for virgin ones (Oehme 1972), *Cloeon* presents an unusual potential for dispersion in mayflies; it is reported even from remote islands such as the Azores in the Northern Atlantic Ocean (Brinck and Scherer 1961), La Réunion in the Indian Ocean (Gattolliat 2004), or Vanuatu in the Pacific Ocean (Gattolliat and Staniczek 2011).

*Cloeon* colonizes all kind of still and standing waters. It can be collected in the riparian vegetation of streams, in ponds and lakes as well as artificial habitats. Larvae feed on detritus and small algae. They swim rather rapidly and actively move their gills. They support water of low quality, i.e. α–β mesosaprobic (Zelinka and Marvan 1961) and even temporary anoxia (Nagell 1977). At least some of the species are recognized as being ovoviviparous [e.g. *Cloeon dipterum* Linnaeus, 1761, *Cloeon smaeleni* Lestage, 1924, *Cloeon gambiae* Gillies, 1980, *Cloeon perkinsi* Barnard, 1932, and *Cloeon cylindroculum* (Kimmins, 1956) (Degrange 1959, Gillies 1985)] implying a long imaginal stage necessary for the maturation of the eggs.

The genus *Cloeon* is characterized at the larval stage by double rounded gills on segments I to VI and gills VII simple; sclerotized spines on the lateral margins of the abdomen; segment III of labial palp apically tapered or falcate; legs long and slender, tarsal claws elongated with two rows of numerous denticles; median caudal filament equal to the cerci. In the imaginal stage: forewing with single intercalary veins; hindwings absent; female forewing with costal and subcostal fields coloured in some species; male with 3-segmented gonopods without lateral extensions, segment III reduced, genital plate rounded or conical.

Historically, several species of *Cloeon* were described from the Western Hemisphere, but all of them have been transferred to other genera. While the species from the Neartic realm were transferred to *Centroptilum* Eaton, 1869 or *Procloeon* Bengtsson, 1915 (McCafferty and Waltz 1990), those from the Neotropics were all transferred to other genera mainly *Pseudocloeon* Klapalek, 1905 - and subsequently to *Americabaetis* Kluge, 1992 (Ulmer 1920, Gillies 1990, Lugo-Ortiz and McCafferty 1999), or to *Callibaetis* Eaton, 1883 (Gillies 1990).

The case of *Cloeon dipterum* (mentioned as *Cloeon cognatum* Stephens, 1835 in some papers) is of great interest; this widespread European species was first reported from the U.S.A. based on a single female from Illinois (Burks 1953), but in the succeeding years additional records were provided from several areas in U.S.A. and Canada (Traver 1962, Flowers 1978, Burian and Gibbs 1991, Randolph et al. 2002, McCafferty et al. 2008). Its presence in North America has long been controversial, with some authors regarding the species as a non-native mayfly (Traver 1962, McCafferty 1996), while others sustaining an old Holarctic distribution (Randolph et al. 2002). Therefore, since 1990, *Cloeon dipterum* is considered the only representative of the genus in the Western Hemisphere where it is restricted to temperate North America.

Recently, unexpected adults and larvae of *Cloeon* were found in the State of Espírito Santo, Southeastern Brazil. During the last seven years this state was one of the most sampled and studied areas in Brazil regarding mayflies (e.g., Salles et al. 2010), including some of the sites where *Cloeon* has been found now. This fact, along with the previous absence of report of true *Cloeon* in South America (Domínguez et al. 2006) and the ecology of the genus (i.e., tolerance to low water quality, ovoviparity), indicates a recent introduction of a non-native species. In order to verify this hypothesis, our aim is to identify the species based on morphological and molecular characters and to confirm the presence of true representatives of the genus in the Neotropics. A brief discussion on the significance of this finding to the origin of *Cloeon dipterum* in North America is also provided, as well as the problems that may arise from the colonization of this taxon in Brazil.

## Material and methods

Larvae were collected with the usual techniques for aquatic insects, such as surber samples and other net sampling methods. *Cloeon* female adults were gathered mostly inside houses, while male adults were obtained by rearing larvae in the laboratory. The examined material is housed in the Coleção Zoológica Norte Capixaba, Universidade Federal do Espírito Santo, São Mateus, Brazil; Coleção Entomológica Prof. José Alfredo Dutra, Universidade Federal do Rio de Janeiro, Rio de Janeiro, Brazil; and Musée cantonal de zoologie, Lausanne, Switzerland. The geographic records of the species were mapped with DIVA-GIS (version 7.17.2, http://www.diva-gis.org/) and then edited with Adobe Illustrator and Adobe Photoshop CS6.

For the specific identification, the most important morphological characters of the genus were examined (e.g. Gillies 1980, Gattolliat and Rabeantoandro 2002, Gattolliat et al. 2008): for the immature stage, the shape of the labial palp, the number of segments of the maxillary palp, the number of abdominal segments with lateral spines and the number of lateral spines, and the shape and denticulation of the tarsal claws; for the imagos, the abdominal color pattern, the shape of the genital plate, the coloration of the costal and subcostal fields of female fore wings. As no identification key for all the species of *Cloeon* are available, several papers were consulted, such as: Gillies (1980, 1985); Gattolliat and Rabeantoandro (2002) for Afrotropical species; Sowa (1975), Bauernfeind and Humpesch (2001), Bauernfeind and Soldán (2012) for Palaearctic species.

For the molecular analyses DNA was extracted from specimens stored in pure alcohol using a Qiagen Extraction Kit. The 658 pb of a mitochondrial protein-coding gene fragment (cytochrome oxydase subunit I, or CO1) were amplified using primers LCO 1490 (GGTCAACAAATCATAAAGATATTGG) and HCO 2198 (TAAACTTCAGGGTGACCA A AAAATCA) (Folmer et al. 1994). All laboratory procedures, edition and alignment of sequences were conducted as described in Vuataz et al (2011). The sequence divergence between haplogroups was calculated using the Kimura 2-parameter as implemented in Mega 5.05 (Tamura et al. 2011). We considered 3% sequence divergence (K2P=0.03) as the maximal value for intraspecific divergence (Hebert et al. 2003). GenBank accession numbers are provided for the material in which DNA has been analysed.

## Material examined

### Cloeon smaeleni

**Brazil**

**Conceição da Barra**, Parque Estadual de Itaúnas, Alagado, 17/v/2012, 18°24'34.66"S, 39°41'59.84"W, 54 larvae, 1 female subimago, 1 female imago, 3 male subimagos and 2 male imagos. Same data, 18/v/2012, 9 larvae (GenBank accession numbers: HG935106 and HG935107). **São Mateus:** Meleira, 17/vi/2011, 18°43'14.8692"S, 39°46'9.1302"W, 2 female imagos; Bairro Colina, 09/x/2012, 1 male imago; Chácara do Cricaré, Rua Traíra, 17 female imagos, 07/vi/2013, 18°42'57.9162"S, 39°50'29.5902"W, 4 female imagos; Rio Preto, 31/x/2012, 18°44'8.16"S, 39°47'47.0394"W, 4 larvae; Rio Preto, 25/ix/2012, 18°44'8.16"S, 39°47'47.03"W, 3 female imagos. **Jaguaré:** Córrego Água Limpa, 04/viii/2012, 18°55'40.3962"S, 39°59'9.8988"W, 7 larvae; Santa Maria, Cachoeira do Bereco, 22/ix/2011, 18°53'4.45"S, 40°12'23.14"W, 2 larvae. **Vitória**, Port Complex of Tubarão: impoundment on Carapina stream, 20°15'23.09"S, 40°14'57.95"W, 15.x.2009, 108 larvae. Same data, 16–17.xii.2009, 5 larvae. Impoundment on Carapina Stream, 20°15'45.89"S, 40°15'1.93"W, 15.x.2009, 7 larvae. Same data, 16–17.xii.2009, 9 larvae. Impoundment on Carapina Stream, 20°15'32.71"S, 40°15'34.29"W, 14.x.2009, 4 larvae. Same data, 16–17.xii.2009, 54 larvae. **Guarapari**, Parque Estadual Paulo César Vinha: Lagoa Feia, 03/v/2012, 19°26'33.71"S, 40°24'7.2"W, 8 larvae; Lagoa Manilha, 02/v/2012, 19°23'40.92"S, 40°25'20.27"W, 3 larvae. **Bom Jesus do Norte**, Ilha do Vicente, Rio Itabapoana, 31/vii/2012, 21°6'53.59"S, 41°41'30.90"W, 2 larvae.

**Madagascar**

Antananarivo, Atanandrano, 23/v/2003, 25 larvae. (GenBank accession numbers: HG935104 and HG935105).

**South Africa**

S2125, Limpopo Prov., Louis Trichardt, Bass. Limpopo, Riv. Luvuvhu, Alt. 700m, 24/v/2003, 23°05'11"S, 30°10'29"E, 15 larvae.

### *Cloeon* cf. *smaeleni*

**Saudi Arabia**

AR47, Al-Itnayn, dam, Alt. 2300m, 14/xi/2012, 18°01'21"N, 42°45'50"E, 10 larvae, 5 female imagos and subimagos. (GenBank accession numbers: HG935108 and HG935109).

### Cloeon dipterum

**Switzerland**

Zurich, Kleinandelfingen, Räubrichsee, 15/v/2012, 47°36'46"N, 8°40'35"E, 2 larvae. (Unpublished sequences from Sereina Rutschmann, IGB, Berlin).

**Korea**

(GenBank accession number: KC135930).

### *Cloeon* cf. *dipterum*

**Canari Islands**

GC01: Gran Canaria, Telde, Barranco de los Cernicalos, 25/i/2009, 27°57'54"N, 15°29'46"W, 6 larvae. (GenBank accession numbers: KF438141 and KF438144).

TF03: Tenerife, Igueste de St Andrés, Alt. 100m, 18/iii/2007, 28°32'21"N, 16°09'26"W, 20 larvae. (GenBank accession numbers: KF438163 and KF438120).

### Cloeon peregrinator

**Madeira**

Funchal, Alt. 270m., 17/iv/2006, 32°39'43"N, 16°53'44"O, 27 larvae

Funchal, Alt. 70m., 02/xii/2005. 32°38'30"N, 16°55'36"O, 2 female imagos.

### *Cloeon praetextum* (gr. *simile*)

**Norway**

Finnmark (GenBank accession number: PRJNA37833).

### *Cloeon* sp1

**Saudi Arabia**

AR44, Wadi Shahadan, Alt. 190m, 13/ii/2012, 17°27'7"N, 42°42'49"E, 20 larvae. (GenBank accession number: KF438120).

### Cheleocloeon soldani

**Saudi Arabia**

AR39, Wadi Damad, Alt. 260m, 11/ii/2012, 17°27'7"N, 42°42'49"E, 35 larvae. (GenBank accession number: HG935111).

## Results

**Figures 1–5.**
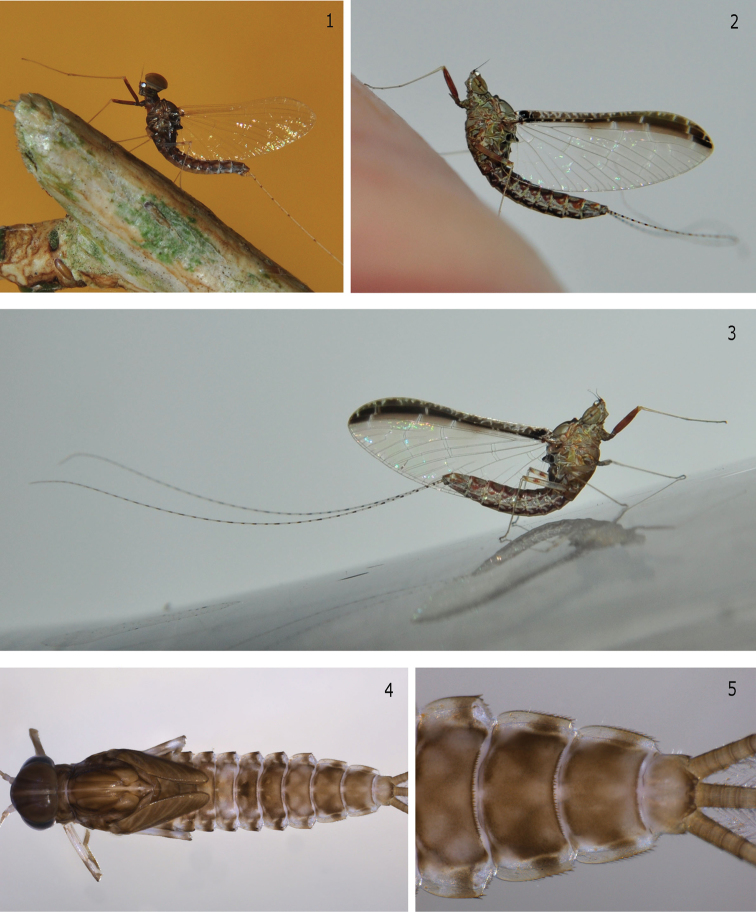
*Cloeon smaeleni*. **1** Male imago (lateral view of living specimen) **2** Female imago (lateral view of living specimen) **3** Female imago (lateral view of living specimen) **4** Male larva (dorsal view) **5** Detail of male larval tergites VII to X.

**Figures 6–17.**
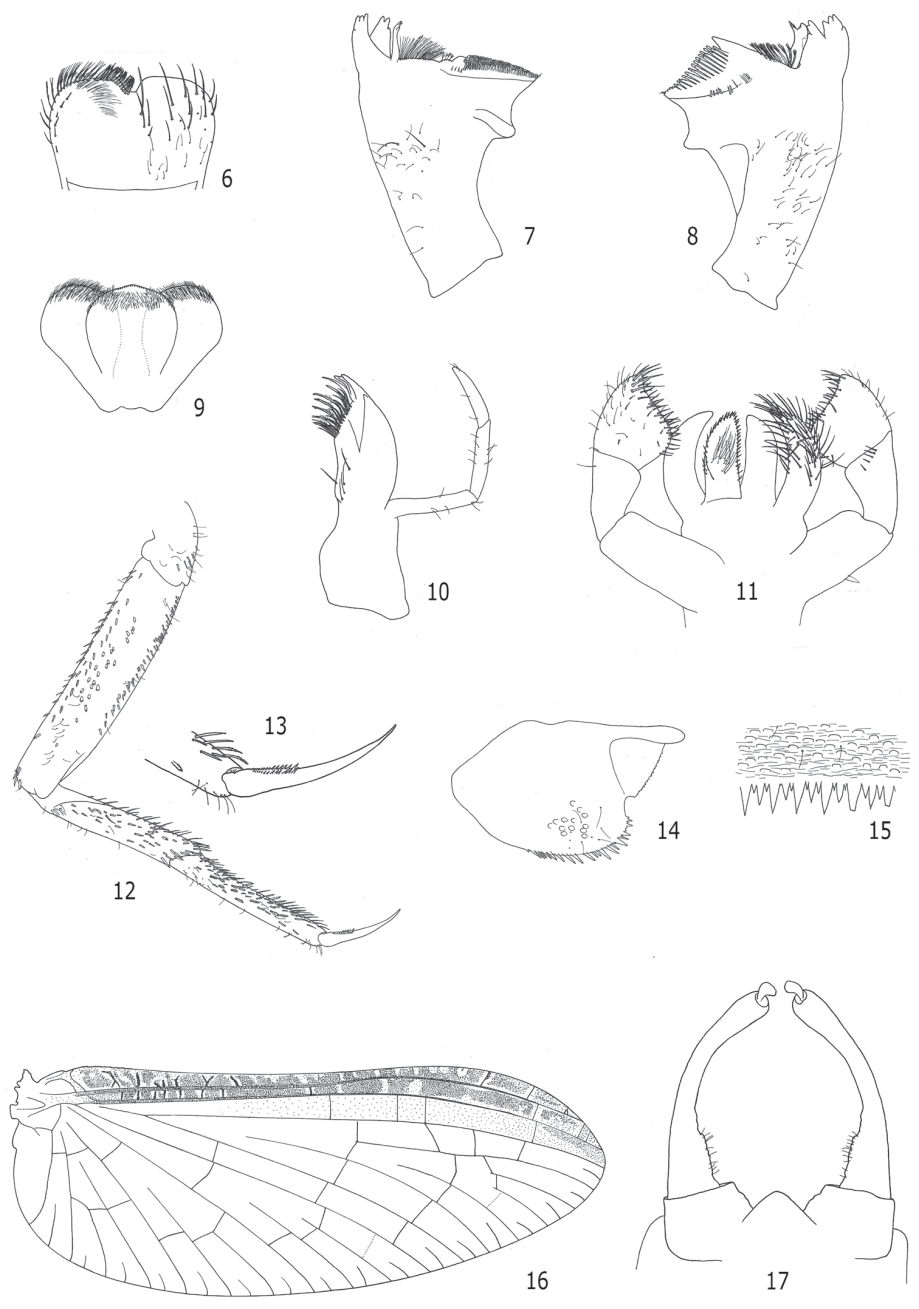
*Cloeon smaeleni*: **6–15** larva morphology **6** Labrum **7** Right mandible **8** Left mandible **9** Hypopharynx **10** Maxilla **11** Labium **12** Fore leg **13** Fore tarsal claw **14** Paraproct **15** Posterior margin of abdominal tergum **16** and **17** adult morphology **16** Female fore wing **17** Male genitalia.

Morphological, as well as molecular analyses revealed that the specimens found in Brazil belong to the Afrotropical species *Cloeon smaeleni*. The main diagnostic characteristics were, as usual among *Cloeon* species, the color pattern of adults, especially the fore wing (Figs 1, 2, 3 and 16) and abdominal sterna (Figs 1, 2 and 3), along with the color of the fore legs (Figs 1, 2 and 3). In addition, the maxillary palp three-segmented (Fig. 10), the labial palp segment III clavate (Fig. 11), the lateral spines restricted to segments VIII and IX (Figs 4 and 5), the teeth and shape of the tarsal claws (Figs 12 and 13), the spines on posterior margin of abdominal terga alternating one long one short (Fig. 15), and the male genitalia with a conical genital plate (Fig. 17) were crucial for the specific identification. Other morphological features such as labrum (Fig. 6), mandibles (Figs 7 and 8), hypopharynx (Fig. 9), and paraproct (Fig. 14) are also illustrated as they may be useful to separate *Cloeon* from other Neotropical genera especially *Callibaetis* Eaton, 1881 and *Callibaetoides* Cruz, Salles & Hamada, 2013.

There is no genetic distance between the Brazilian specimens and they are highly supported as sister group to Afrotropical haplotypes of *Cloeon smaeleni* (Table 1). The genetic distance between Brazilian and Afrotropical haplotypes clearly corresponds to intraspecific variation (K2P distance = 0.02). We also sequenced material from Saudi Arabia morphologically similar to *Cloeon smaeleni*. These haplotypes appear as sister group to the Afrotropical + Brazilian clade, but present interspecific distance with this clade (K2P distance > 0.11). Brazilian haplotypes are not genetically related to any Palaearctic species including *Cloeon dipterum* *s. l.* (K2P distance > 0.20).

**Table 1.** Sequences using the Kimura 2-parameter: **Taxa:** CS = *Cloeon smaeleni* or *Cloeon* cf. *smaeleni*; CD = *Cloeon dipterum* or *Cloeon* cf. *dipterum*; CL: *Cloeon* sp1; CP = *Cloeon praetextum*; CHS = *Cheleocloeon soldani*. **Countries:** MA = Madagascar; BR = Brazil; SA = Saudi Arabia; CH = Switzerland; KO = South Korea; TF = Tenerife (Canari Islands); GC = Gran Canaria (Canari Islands); NO = Norway.

	CS-MA	CS-BR	CS-SA	CD-CH	CD-KO	CD-TF	CD-GC	CL-SA	CP-NO
**CS-BR**	0.02								
**CS-SA**	0.12	0.11							
**CD-CH**	0.19	0.20	0.18						
**CD-KO**	0.21	0.21	0.19	0.08					
**CD-TF**	0.21	0.22	0.20	0.10	0.09				
**CD-GC**	0.22	0.23	0.21	0.11	0.10	0.10			
**CL-SA**	0.19	0.19	0.20	0.17	0.17	0.18	0.20		
**CP-NO**	0.23	0.23	0.21	0.21	0.20	0.21	0.20	0.19	
**CHS-SA**	0.24	0.25	0.23	0.20	0.20	0.22	0.23	0.21	0.23

**Figures 18–21.**
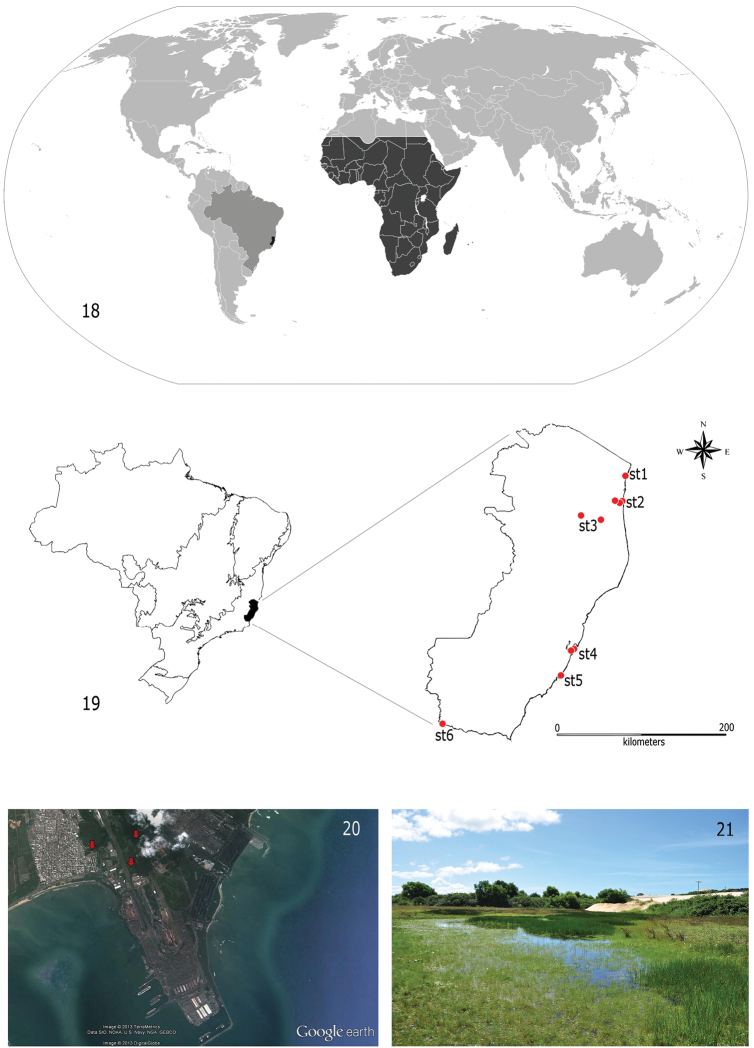
Distribution of *Cloeon smaeleni*. **18** World map (gray, Brazil; dark gray, species distribution) **19** Map of Brazil subdivided in biomes, with detail of the State of Espírito Santo and collection stations (red circles) (st1, Conceição da Barra; st2, São Mateus; st3, Jaguaré; st4, Vitória; st5, Guarapari; st6, Bom Jesus do Norte) **20** Satellite picture from the Tubarão Complex Port in Vitória (red arrows indicate collection stations) **21** General view of one of the stations at Parque Estadual de Itaúnas.

The specimens examined were found exclusively in the State of Espírito Santo, Southeastern Brazil. They have been reported from at least six localities, most of them along the coast of the state (Figs 18 and 19) and always in low altitude areas (from the sea level to 65 meters above it). In Vitória (st4), Guarapari (st5) and Conceição da Barra (st1) (Fig. 19), larvae were collected in ponds very close to the shoreline. In Vitoria (st4), larvae were gathered in artificial impoundments colonized by *Pistia* spp. or *Typha* spp. macrophytes on the final section of the Carapina stream. All impoundments are located inside the Port Complex of Tubarão (Fig. 20), the biggest iron ore export port in the world. In São Mateus (st2), larvae were found in Rio Preto, a small black water tributary (Fig. 19) of the main river of the region, the Rio São Mateus or Cricaré. Female adults were caught inside a house very close to a large tributary of the Cricaré, the Rio Mariricu, suggesting that the species might also be present there. Attempts to collect material at the Rio Cricaré, however, were unsuccessful. Jaguaré (st3) and Bom Jesus do Norte (st6) are located more distant from the Ocean coast (around 100 km); in these localities, specimens were collected in approximately five meter wide streams. In the streams or rivers, larvae were found exclusively in areas with slow or no current, among organic substrates, such as roots, macrophytes or leaf litter.

## Discussion

*Cloeon smaeleni* is a widespread species (Fig. 18) originally described from a female imago from Katanga, Congo. Subsequently, all ontogenetic stages of *Cloeon smaeleni* were described (Gillies 1980, Gattolliat and Rabeantoandro 2002) and this species was reported from the whole Afrotropical Region: Central Africa (Lestage 1924), Southern Africa (Demoulin 1970), West Africa (Gillies 1980), East Africa (Gillies 1985), Madagascar (Gattolliat and Rabeantoandro 2002), and La Réunion (Gattolliat 2004). *Cloeon smaeleni* was also reported from the Arabian Peninsula (Gillies 1985). Our molecular (Table 1) and morphological analyses (unpublished results) established that in fact Arabian populations constitute a new undescribed species related to *Cloeon smaeleni*.

Larvae of *Cloeon smaeleni* are found in many types of slow waters: temporary ponds, rice fields, reservoirs, slow moving streams and the margins of lakes (Gillies 1980), sometimes associated with hydrophytes (Petr 1968). Imagos, as well as other species of the genus, are ovoviviparous (Gillies 1985).

The identity of the species, its distribution, along with its previous absence in regularly sampled sites, is a clear sign that the specimens of *Cloeon smaeleni* found in Espírito Santo are introduced, well established, and that the colonization took place very recently. It is not possible to ascertain where or how this event has occurred, or even if there was a single or multiple entrances. The presence of larvae of *Cloeon smaeleni* in a port, though highly speculative, suggests that they may have arrived in by ship traveling from Africa.

It is also difficult to predict the impact caused by the presence of *Cloeon smaeleni* in Brazil. In its original habitats, this species feeds on detritus contributing to the recycling of organic matter. It is often the eudominant species but generally co-occurs with other *Cloeon* species. As this species is not a predator and has no significant economic importance, the impact is probably more related to its competition with other native species at the same trophic level (or controphics species, according to Davis 2003). In the case of the mayfly community, species of the genus *Callibaetis* occupy a very similar ecological niche when compare to those of *Cloeon* (Domínguez et al. 2006). *Callibaetis guttatus* Navás, 1915, for example, is found along the Brazilian tropical coast (Da Silva 1991, Salles et al. 2010, Lima et al. 2012) and is a common species in the sites where *Cloeon smaeleni* was collected. Based on our observations in the north of Espírito Santo, at least for now, larvae of *Callibaetis* are more abundant than those of *Cloeon*. Following Davis (2003), competition from introduced species is not likely to be a common cause of extinctions of long-term resident species. However, a study on the population dynamics of both species would be interesting in order to monitor the potential impact of *Cloeon smaeleni* on a resident species, especially because the sites where the species was found in Conceição da Barra (st1) and Guarapari (st5) are located in nature reserves, the Parque Estadual Paulo Cesar Vinha and the Parque Estadual de Itaúnas (Fig. 21).

Our finding raises again the idea, put forward by earlier authors (e.g. Traver 1962, McCafferty 1996, McCafferty and Mauremootoo 2001), but contested by Randolph et al. (2002), that *Cloeon dipterum* is also an exotic species in the Western Hemisphere. Whereas *Cloeon smaeleni* is a tropical species that encountered a suitable area for reproduction along the coast of Brazil, similar to its native habitat, the same is also plausible for *Cloeon dipterum* in North America. This result also has important implication on the insular populations of *Cloeon* that are often considered as endemics but may be in fact recent natural colonizations or introductions related to human activities (McCafferty and Mauremootoo 2001, Gattolliat and Staniczek 2011, Gattolliat 2013). On the other hand, *Cloeon dipterum* is not the only species of mayfly, or even Baetidae, with a Holarctic distribution. Kjaerstad et al. (2012), for example, listed 10 species found in Norway and North America. Besides these species, the CO1 sequences of *Cloeon praetextum* Bengtsson, 1914 studied by these authors are highly similar to those of *Procloeon mendax* (Walsh, 1862) from northern Canada (less than 1%) and, therefore, they maybe conspecific. The historical processes that lead to the presence of unlikely invasive species in both realms, such as those listed by Kjaerstad et al. (2012), could be the same that lead to the Holarctic distribution of *Cloeon dipterum*.
